# Force Plate with Simple Mechanical Springs and Separated Noncontact Sensor Elements

**DOI:** 10.3390/s21217092

**Published:** 2021-10-26

**Authors:** Yuta Kawasaki, Ami Ogawa, Hidetoshi Takahashi

**Affiliations:** 1 Department of Mechanical Engineering, Faculty of Science and Technology, Keio University, 3-14-1 Hiyoshi, Kouhoku-ku, Yokohama 223-8522, Kanagawa, Japan; yuta.kawa@keio.jp; 2 Department of System Design Engineering, Faculty of Science and Technology, Keio University, 3-14-1 Hiyoshi, Kouhoku-ku, Yokohama 223-8522, Kanagawa, Japan; ogawa@sd.keio.ac.jp

**Keywords:** ground reaction force, force plate, noncontact distance sensor

## Abstract

This paper reports on a force plate (FP) using mechanical springs and noncontact distance sensors. The ground reaction force (GRF) is one of the factors for clarify biomechanics, and FPs are widely used to measure it. The sensor elements of conventional FPs are mainly strain gauges. Thus, the mechanical properties of FP depend on the sensor element performance. If the FP performance must change, we must redesign the FP, including changing the sensor elements. Here, we proposed an FP that uses a measuring principle based on simple springs and noncontact sensors. The shape and performance of the proposed FP are expected to change easily. As a prototype device, we designed and fabricated an FP installed with 12 springs and four sensors for human walking. A planar coil and magnet were used as the sensor elements, and the sensor output was proportional to the vertical and horizontal displacements. The FP resonance frequency was 123 Hz, which was larger than the required specification. The calibration experiments showed that vertical and horizontal forces and moments could be measured independently. The FP’s resolutions were 1.9 N and 1.4 N in the anterior–posterior and vertical directions, respectively. Furthermore, the fabricated FP measured GRF similarly to the commercial FP when a human walked on the plate. These results suggest that the proposed method will be helpful for FPs with custom-made requirements.

## 1. Introduction

Ground reaction force (GRF) on the sole is one of the factors used to clarify foot biomechanics [1,2]. A significant number of reports have evaluated the GRF using several methods, such as force plates (FPs) [3,4,5,6,7], treadmills [8,9,10], and attaching force sensors to shoe soles [11,12]. Among them, the FP system has been widely used due to its high-accuracy measurement in multiple directions without needing to be worn by the subject. The sensor elements of the conventional FP have normally been of the strain gauge type [13] or load cell type [14,15], which are in direct contact with the plate. The mechanical characteristics of the FP, such as the measurable force range, resonant frequency, and shape, depend on the sensor elements. Thus, the size and dimension of commercialized FPs are basically fixed; it is expensive to change the specifications because then the FP must be redesigned, including the sensor element.

For traditional GRF measurement, the subject walks around a designated area in the laboratory. Therefore, the use of standard size FPs was not a problem. In recent years, several researchers have focused on measurement in living spaces [16] to investigate the mechanism of disease onset [17], falls [18,19,20], or slips for their prevention in older persons [21]. Since the installation space is limited in living spaces, it is necessary to place an FP that fits each space for the GRF measurement. In particular, stair dimensions, such as the riser height and run length, are known to affect the stair walking performances [22,23,24,25,26]. Flexible resizing is required for the FP to realize GRF measurements under various stair dimensions. Thus, it is advisable to tune the FP’s shape to be suitable for the situation. Additionally, we must change the shape and force range when we measure animals other than humans [27,28]. However, conventional FP components are not suitable for freely changing the performance, including the shape and force range.

Here, we propose an FP, the performance of which can be easily modified according to the application, such as for indoor or target subjects. The proposed FP is composed of a plate with supporting mechanical springs and noncontact displacement sensor elements. By measuring the deflection of the springs with the sensor element, it is possible to measure the force applied to the FP. The sensor element is mechanically separated from the plate; thus, it does not affect the mechanical properties of the FP. Therefore, when the FP must be redesigned in terms of the shape or performance, we can simply redesign the mechanical structure of the plate and springs.

The study aims to prove the practical applicability of the proposed FP measurement method. Thus, in this paper, we first introduce the principle and design of our proposed FP. Second, we evaluate the mechanical characteristics of the designed FP using simulation. Then, we conduct calibration experiments using the fabricated FP. Finally, we demonstrate GRF measurement with the fabricated FP and commercial FP.

## 2. Design and Principle

### 2.1. Sensor Design

To realize the GRF measurement via an FP, three significant specifications are required: the plate dimensions, measurable force direction and range, and time resolution. As the measurement target, the FP was designed to measure the GRF during walking on indoor corridors or stairs. Thus, the plate was first designed to be similar to residential stairs in depth (200 mm) so that a single foot lands on the plate surface. Second, we selected the gravity and horizontal directions as the measurable directions. Previous studies reported that the maximum GRF while walking stairs and corridors in the gravity and horizontal directions became approximately 160% and 20% of the body weight, respectively [29,30]. Thus, assuming that the body weight is 50 kg, the force range should be over 8.0 × 10^2^ N and 1.0 × 10^2^ N in the gravity and horizontal directions, respectively. Previous research has also reported that a resonant frequency of over 100 Hz is sufficiently high to accurately measure the GRF [31]. Thus, we defined the specifications of the force plate to satisfy these force ranges and resonance frequencies.

**Figure 1:**
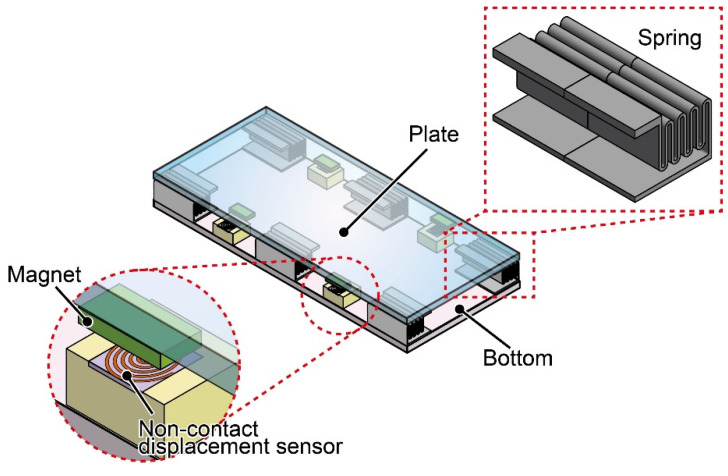
Schematic image of the force plate. The force plate is composed of mechanical springs and noncontact displacement sensor elements using a planar coil.

A schematic image of the proposed FP is shown in Figure 1. The FP consists of a plate, a bottom, 12 mechanical springs, and four sensor elements that work as noncontact two-dimensional displacement sensors. The plate is supported by springs fixed to the bottom. These springs are designed to easily deform in the gravity and horizontal directions. As the sensor element, a magnet and planar coil set are fixed to the plate and bottom, respectively, to measure the noncontact plate displacement. When walking on the plate surface, each sensor element measures the local two-dimensional displacements of the plate due to the expansion and contraction of the springs. Then, the force can be calculated from the sensor outputs.

The resonant frequency, force range, and plate geometry can be tuned without changing the sensor element. Thus, a high degree of freedom in design is possible. Meanwhile, we should pay attention to the following point. The low stiffness of the plate section and high stiffness of the spring increase the FP error, where the force is applied, because the deflection of the plate prevents an accurate measurement of the plate displacement by spring deformation. The high mass of the plate and low spring constant reduce the resonant frequency of the FP. Therefore, the plate should be light and highly rigid, and the spring should be designed with a spring constant that considers the positional error and resonant frequency.

### 2.2. Force Detection Principle

In this study, we used noncontact displacement sensors with a planar coil and magnet sheet as the sensing elements. A planar coil 10 mm in diameter was used. When the positional relationship between the planar coil flowing AC and the magnet changes, the output frequency changes due to magnetic field changes. The displacement is measured by converting the frequency change into a voltage change.

**Figure 2:**
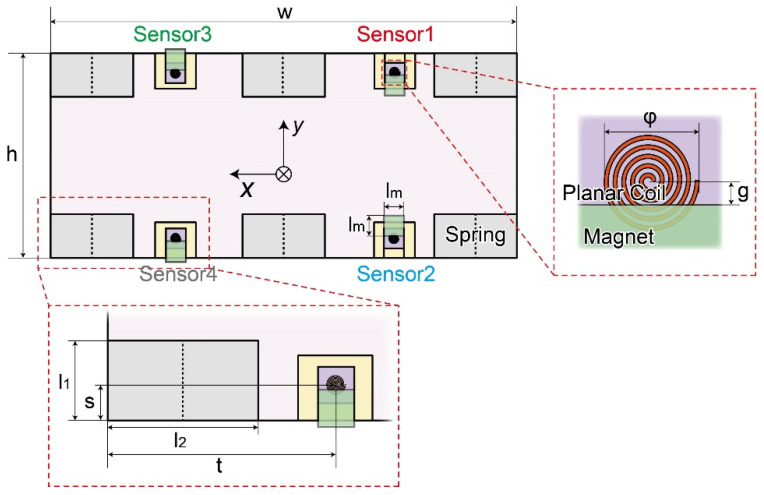
Schematic image of the position and dimension of the force plate components.

The sensor measures two-directional displacements of the plate by fixing the magnet sheet to the plate backside to half-overlap with the planar coil, as shown in Figure 2. The output voltage of the planar coil decreases when the magnet is close to the planar coil. In the opposite case, the output voltage increases. Due to the half-overlapping relation, the output changes by both vertical and horizontal displacements.

Table 1 shows the position and size dimensions of the coils, magnets, springs, and plate. The height *h* and width *w* of the force plates were 200 mm and 400 mm, respectively. The height *l*_1_ and width *l*_2_ of the spring were 43 mm and 80 mm, respectively. The position of the coil is 24 mm away in length *s* and 120 mm away in width *t* from the edge. The initial distance *g* between the center of the coil and the edge of the magnet is 3 mm when no force is applied to the force plate. We numbered the sensor element from “Sensor 1” to “Sensor 4”, as shown in Figure 2.

When *F*_z_ is applied, all magnets approach the planar coils so that all outputs become low. When *F*_y_ is applied, the outputs of sensors 1 and 4 become high because the magnet moves away from the coil, while sensors 2 and 3 become low because they approach each other. When *F*_x_ is applied, the positional relationship between the coils and the magnets does not change, and the spring deforms little in the *x*-direction due to the high stiffness, so that no outputs change. When *M*_x_ or *M*_y_ is applied, the plate inclines, and *z*-direction displacement occurs. When *M*_x_ is applied, the outputs of sensors 4 and 2 become high because the magnet moves away from the coil, while sensors 1 and 3 become low because they approach each other. When *M*_y_ is applied, sensors 3 and 4 become high, while sensors 1 and 2 become low. When *M*_z_ is applied, the plate rotates horizontally so that the outputs of sensors 1 and 3 become high, while sensors 2 and 4 become low. Thus, the combination of positive and negative sensor output changes is identical to that when *M*_x_ is applied. The measured *M*_x_ in normal walking is approximately four times as large as *M*_z_ [32]. In addition, the plate displacement when Mz is applied to FP is approximately one-tenth of that when *M*_z_ is applied. Therefore, the sensor output caused by *M*_z_ is negligible. Hence, the force in each direction can be measured with good independence by the positive and negative sensor outputs, as shown in Table 2.

Assuming that displacement is only caused by the springs’ deformation—that is, the plate is an undeformable rigid body—we can evaluate the force by the linear sum of the plate displacement. Furthermore, assuming that the sensor output is proportional to the displacement of the plate, the force can be evaluated as a linear sum of the sensor outputs. Then, the force matrix is expressed by calibration matrix ***B*** as follows:

(1) $$\begin{array}{c} \left[\begin{array}{c} F_{y}\\ F_{z}\\ M_{x}\\ M_{y} \end{array}\right]=\left[\begin{array}{cccc} 0 & 0 & 0 & 0\\ B_{1F_{z}} & B_{2F_{z}} & B_{3F_{z}} & B_{4F_{z}}\\ B_{1M_{x}} & B_{2M_{x}} & B_{3M_{x}} & B_{4M_{x}}\\ B_{1M_{y}} & B_{2M_{y}} & B_{3M_{y}} & B_{4M_{y}} \end{array}\right]\left[\begin{array}{c} \Delta l_{z1}\\ \Delta l_{z2}\\ \Delta l_{z3}\\ \Delta l_{z4} \end{array}\right]+\left[\begin{array}{cccc} B_{1F_{y}} & B_{2F_{y}} & B_{3F_{y}} & B_{4F_{y}}\\ 0 & 0 & 0 & 0\\ 0 & 0 & 0 & 0\\ 0 & 0 & 0 & 0 \end{array}\right]\left[\begin{array}{c} \Delta l_{y1}\\ \Delta l_{y2}\\ \Delta l_{y3}\\ \Delta l_{y4} \end{array}\right]\\ =\left[\begin{array}{cccc} B_{1F_{y}} & B_{2F_{y}} & B_{3F_{y}} & B_{4F_{y}}\\ B_{1F_{z}} & B_{2F_{z}} & B_{3F_{z}} & B_{4F_{z}}\\ B_{1M_{x}} & B_{2M_{x}} & B_{3M_{x}} & B_{4M_{x}}\\ B_{1M_{y}} & B_{2M_{y}} & B_{3M_{y}} & B_{4M_{y}} \end{array}\right]\left[\begin{array}{c} \Delta l_{1}\\ \Delta l_{2}\\ \Delta l_{3}\\ \Delta l_{4} \end{array}\right]=Bl \end{array},$$

where Δ*l*_z1_, Δ*l*_z2_, Δ*l*_z3_, and Δ*l*_z4_ are the *z*-directional displacements of the plate at four points, and Δ*l*_y1_, Δ*l*_y2_, Δ*l*_y3_, and Δ*l*_y4_ are the *y*-directional displacements of the plate at four points. If there is nonlinearity in the sensitivity, measurement error may be caused by the matrix. These two displacements are defined as Δ*l*_1_, Δ*l*_2_, Δ*l*_3_, and Δ*l*_4_ to simplify the equation. Assuming that the sensor output is proportional to the displacement of the plate, the force can be calculated using the following equation with calibration matrix ***A***:

(2) $$\begin{array}{c} \left[\begin{array}{c} F_{y}\\ F_{z}\\ M_{x}\\ M_{y} \end{array}\right]=\left[\begin{array}{cccc} A_{1F_{y}} & A_{2F_{y}} & A_{3F_{y}} & A_{4F_{y}}\\ A_{1F_{z}} & A_{2F_{z}} & A_{3F_{z}} & A_{4F_{z}}\\ A_{1M_{x}} & A_{2M_{x}} & A_{3M_{x}} & A_{4M_{x}}\\ A_{1M_{y}} & A_{2M_{y}} & A_{3M_{y}} & A_{4M_{y}} \end{array}\right]\left[\begin{array}{c} \Delta V_{1}\\ \Delta V_{2}\\ \Delta V_{3}\\ \Delta V_{4} \end{array}\right]\\ =AV \end{array}.$$

Equation (2) shows that the force can be obtained as a linear sum of the sensor outputs Δ*V*_1_, Δ*V*_2_, Δ*V*_3_, and Δ*V*_4_. In an ideal FP, the plate is entirely rigid; the plate deflection does not affect the results of the displacement sensors. However, in practice, there is the possibility that the deflection of the plate is added to the displacement measured by the four displacement sensors. Additionally, the plate deflection depends on the position where the force is applied.

It is necessary to consider the position error caused by the plate deflection when calculating the force using Equation (1). The calibration method is described below. The plate is divided into several blocks, and the center of each block is defined as position *k*. When a force is applied at position *k*, the real force *F*_z_ is represented by the calculated force *F*_z*k*_ and error *ε*_z*k*_:

(3) $$F_{z}=F_{zk}+\varepsilon_{k}=\sum_{i=1}^{4}B_{iF_{z}}\Delta l_{ik}+\varepsilon_{zk}.$$

Then, if $\Delta l_{ik}/F_{z}$ is defined as *S*′_z*ik*_, Equation (3) is transformed as follows:

(4) $$\frac{\varepsilon_{k}}{F_{z}}=1-\sum_{i=1}^{4}B_{iF_{z}}{S^{\prime }}_{zik}.$$

For all positions, the sum of the squares of the errors can be expressed as follows:

(5) $$\sum_{k}{\left(\frac{\varepsilon_{k}}{F_{z}}\right)}^{2}=\sum_{k}{\left(1-\sum_{i=1}^{4}B_{iF_{z}}{S^{\prime }}_{zik}\right)}^{2}.$$

When *S*′_z*ik*_ is obtained, the least-squares method is applied to obtain the calibration matrix element *B_i_*_Fz_ that minimizes the squared sum of the errors at all positions. Equations (3)–(5) are calculated for *F*_z_. In addition, the same calculation can be applied to *F*_y_ and moments. As a result, the error range of the measured value is quantitatively ensured regardless of where the force is applied.

**Figure 3:**
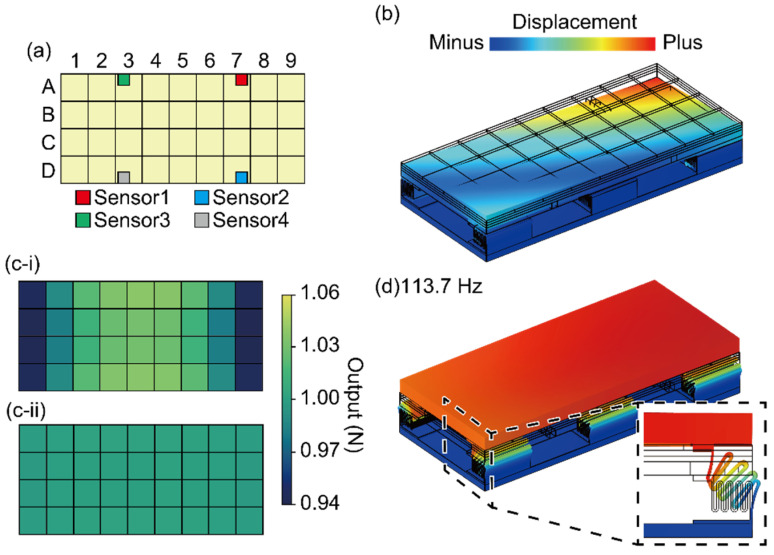
(**a**) Schematic image of the divided area. (**b**) Simulation result of the deformation when *F_z_* is added to A9. Position error in the (**c-i**) *z*-direction and (**c-ii**) *y*-direction. (**d**) Simulation result of the first resonant frequency.

We evaluated the position error of the FP using the finite element method (FEM) (COMSOL Multiphysics v5.5, COMSOL). The plate displacement was simulated when a force was applied to one of the 36 surfaces, which are divided as shown in Figure 3a. For example, when *F*_z_ is applied to A9, the number is defined in Figure 3a, and the FP deforms, as shown in Figure 3b. In this simulation, the FP structure is composed of a plate (carbon; Young’s modulus: 20 GPa) and an aluminum housing (A5052; Young’s modulus: 70 GPa). Other parts are made of stainless steel (SUS304; Young’s modulus: 193 GPa).

We obtained *S_ik_* from the simulation results. The simulated calibration matrix ***B*** obtained using Equation (5) is expressed as follows:

(6) $$B=\left[\begin{array}{cccc} 619 & 693 & 728 & 654\\ 421 & 364 & 469 & 357\\ 33.0 & -34.8 & 36.8 & -34.9\\ 104 & 106 & -109 & -106 \end{array}\right].$$

The units of calibration matrix ***B*** are N/mm for the top two rows and N·m/mm for the bottom two rows. Assuming that the resolution of each displacement sensor is 1.0 μm, the resolutions of the FP are calculated to be *F*_y_: 2.7 N, *F*_z_: 1.6 N, *M*_x_: 0.14 N/m, and *M*_y_: 0.42 N/m.

We simulated the plate displacements when 1 N was applied in each area in the *y*- and *z*-directions. Then, we calculated *F*_y_ and *F*_z_ in each area with Equation (1) using these results and calibration matrix ***B*** in Equation (6). Figure 3c shows the schematic image of *F*_z_ and *F*_y_ calculated in each area. Since there is no error depending on the position of the force applied, *F*_z_ and *F*_y_ should be 1 N in all areas. Thus, the position error is determined by how far the output is from 1 N. The *z*-direction and *y*-direction position errors were less than ±6% and ±0.2%, respectively. Thus, we must calibrate the FP in the *z*-direction by considering the position error, while the positional error in the *y*-direction is sufficiently small compared to the positional error in the *z*-direction.

The resonant frequency was also simulated by FEM. It was 113.7 Hz with the first mode, as shown in Figure 3d; the entire plate of the FP vibrates up and down when it resonates. Thus, the resonance frequency is sufficiently large for the GRF measurement.

## 3. Fabrication and Assembly

### 3.1. Sensing Element

**Figure 4:**
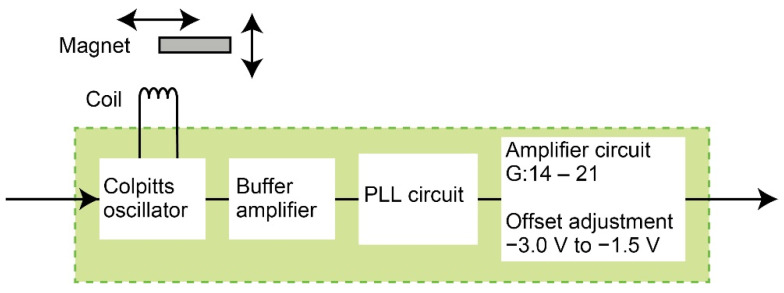
Schematic image of the noncontact displacement sensor.

A schematic circuit image of the displacement sensor is shown in Figure 4. A planar coil whose diameter, resistance, and reactance were 10 mm, 1 Ω, and 8.5 μH was used. An AC voltage of 1.1 MHz was applied to the planar coil via a Colpitts oscillator circuit. Through an amplifier circuit, a phase-locked loop (PLL) circuit converted the output frequency into a voltage.

**Figure 5:**
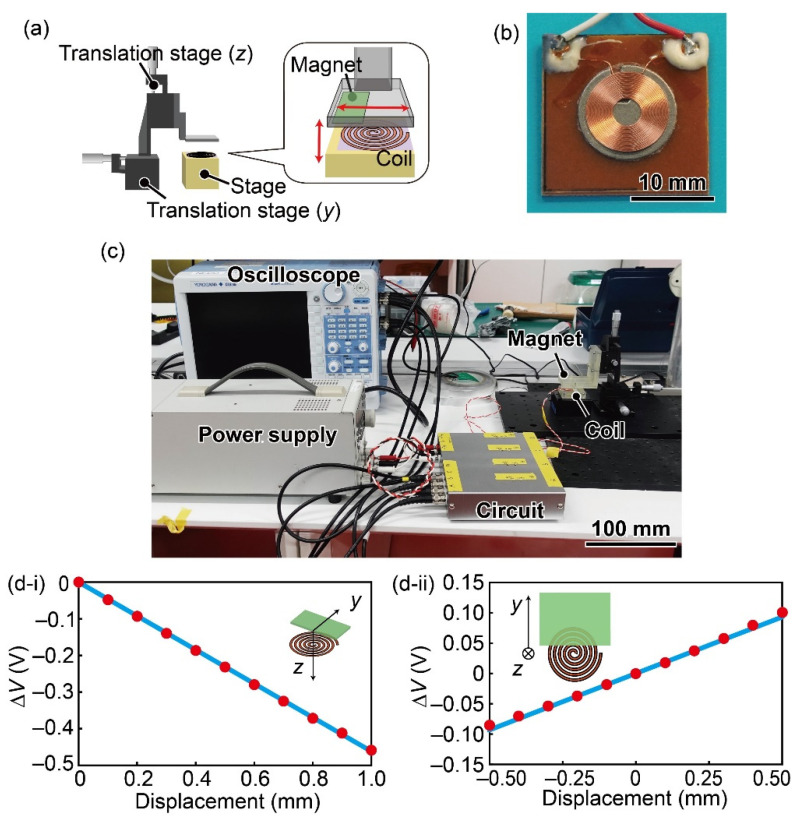
(**a**) Schematic image of the experiment to evaluate the displacement sensor. (**b**) Photograph of the planar coil. (**c**) Photograph of the experimental setup. Relationship between the sensor output and the displacement in the (**d-i**) z-direction and (**d-ii**) y-direction.

We evaluated the relationship between the displacement of the magnet and the output voltage of the developed coil circuit. First, the coil and magnet were attached to be fixed and *z*–*y* translation stages, respectively, as shown in Figure 5a,c. Figure 5b shows a photograph of the planar coil that was used. The *z*-direction origin displacement between the magnet and the planar coil was set to 8 mm; that of the y-direction was set to 3 mm from the coil center, as shown in Figure 2.

The experimental results are shown in Figure 5d. The correlation coefficients between the magnet displacement and the sensor output were −1.00 in the *z*-direction and 1.00 in the *y*-direction. Thus, we confirmed that the displacement and sensor output were proportional to each other. The sensitivity was 5.4 × 10^3^ μm/V and −2.1 × 10^3^ μm/V in the *z*-direction and *y*-direction, respectively. Based on the 80-Hz low-pass-filtered noise data, the resolution was calculated to be 2.9 μm in the *y*-direction and 1.1 μm in the *z*-direction.

### 3.2. Mechanical Spring

**Figure 6:**
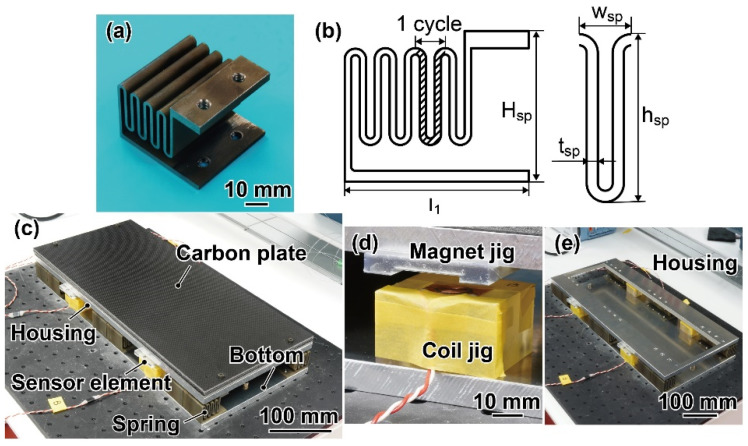
(**a**) Photograph and (**b**) schematic image of the fabricated spring. (**c**–**e**) Photographs of the fabricated force plate, sensor element, and housing.

A photograph and a schematic image of the fabricated spring are shown in Figure 6a,b. This spring has a zigzag pattern of four bumps in the *z*-direction extending in the *y*-direction. The height *H*_sp_ and width *l*_1_ of the spring were 35 mm and 43 mm, respectively. The height *h*_sp_, width *w*_sp_, and thickness *t*_sp_ were 22.5 mm, 7 mm, and 1.5 mm, respectively. The depth was 40 mm (*l*_2_/2 in Figure 2). The spring design structure results in low spring constants in the *z*- and *y*-directions and relatively high spring constants in the *x*-direction due to the large depth relative to *t*_sp_. The spring constant for a single spring, which was simulated by the finite element method, became 1.3 × 10^5^ N/m in the *z*-direction, 2.3 × 10^5^ N/m in the *y*-direction, and 4.5 × 10^5^ N/m in the *x*-direction. Twelve springs were built in the bottom. The spring constant for the FP, simulated by the finite element method, became 1.5 × 10^6^ N/m in the z-direction, 2.9 × 10^6^ N/m in the y-direction, and 1.4 × 10^7^ N/m in the x-direction.

### 3.3. Sensor Assembly

As shown in Figure 6c, the fabricated FP consisted of a carbon plate (ST-LAYER, Sekisui Kasei Co., Ltd., Osaka, Japan), one aluminum housing for plate reinforcement, four sensor elements, 12 springs, and one aluminum bottom. The thicknesses of the carbon plate, housing, and bottom were 12 mm, 10 mm, and 10 mm, respectively. As described in Section 3.1, the sensor elements consist of a planar coil and a neodyminummagnet. The coils and magnets were fixed on the bottom and housing with a jig, as shown in Figure 6d. The coil and magnet jigs were designed and fabricated by a 3D printer (Form3, Formlabs, Somerville, MA, USA), so that the initial distance between them was 8 mm. We made the housing hollow, as shown in Figure 6e. The housing and hollow sizes were 450 mm × 200 mm and 430 mm × 110 mm. The weights of the carbon plate and housing were 0.60 kg and 1.15 kg, respectively. The entire FP size and weight were 200 mm × 450 mm × 67 mm and 6.3 kg, respectively.

## 4. Experiment and Results

### 4.1. Resonant Frequency

**Figure 7:**
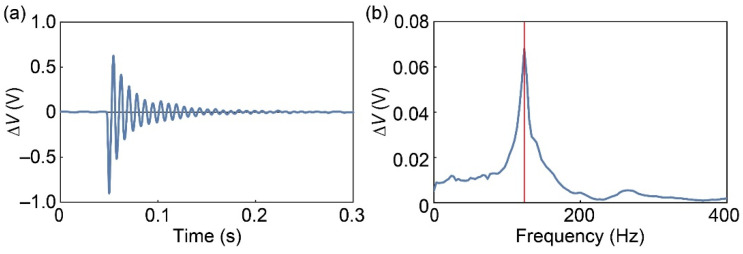
(**a**) Output voltage of sensor 1 when an impulse force is applied. (**b**) Measured resonance frequency.

The resonant frequency of the fabricated FP was evaluated. We impacted the FP center with a hammer and measured the output of sensor 1. Figure 7 shows the output waveform with a 400-Hz low-pass filter and the Fourier transform. As a result, the resonance frequency of the FP was 123 Hz. This value is larger than the required specification of 100 Hz and the simulation result.

### 4.2. Force Calibration

Two calibration experiments were conducted: one applies *F*_z_, and the other applies *F*_y_. First, the calibration experiment applying *F*_z_ is described below. Since the sensor output and displacement are proportional, Equation (2) is valid. Therefore, as in Equation (3), the following equation is valid.

(7) $$F=F_{k}+\varepsilon_{k}=\sum_{i=1}^{4}A_{iF_{z}}\Delta V_{ik}+\varepsilon_{k}.$$

Then, providing that $\Delta V_{ik}/F$ is defined as *S_ik_*, the least-squares method is applied to obtain the calibration matrix element *A_iF_*_z_ that minimizes the squared sum of the errors at all positions and Equations (4) and (5):

(8) $$\sum_{k}{\left(\frac{\varepsilon_{k}}{F}\right)}^{2}=\sum_{k}{\left(1-\sum_{i=1}^{4}A_{iF_{z}}S_{ik}\right)}^{2}.$$

**Figure 8:**
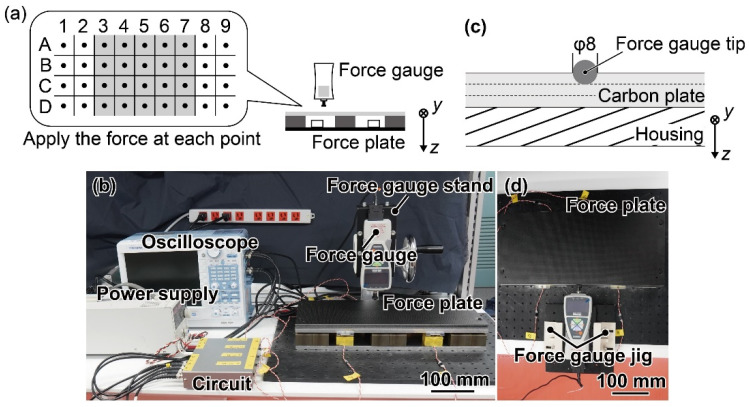
(**a**) Schematic image and (**b**) photograph of the calibration in the *z*-direction. (**c**) Schematic image and (**d**) photograph of the calibration in *y*-direction.

As mentioned in Section 2.2, there was a positional error in the measurement of the *F*_z_ direction. Therefore, the FP was divided into 36 squares of 50 mm square in four rows and nine columns with identical force applied points as those in the simulation (Figure 8a). Assuming that a foot would land around the FP center, we calibrated by applying force only to rows 3–7. Figure 8b shows a photograph of the experimental setup. *F*_z_ was applied to the FP with a force gauge (ZTA-1000N, IMADA Co., Ltd., Aiti, Japan) via a manual test stand (SVH1000N, IMADA Co., Ltd., Aiti, Japan) until over 500 N. The manual test stand enabled the force gauge to move only in the *z*-direction. *F*_z_ was applied to each point by moving the FP. Then, the outputs of the force gauge and each sensor element were simultaneously recorded by an oscilloscope (DL850, Yokogawa Electric Corporation, Tokyo, Japan). When *F*_z_ was applied to a point other than the center of FP, a moment was applied to FP. Therefore, from the experimental results, it was possible to evaluate the calibration matrices for *F*_z_, *M*_x_, and *M*_y_.

We also conducted a calibration experiment with *F*_y_. The experimental conditions of the oscilloscope, circuit, and stabilized power supply were identical to those for *F*_z_. As mentioned in Section 2.2, *F*_y_ has a smaller positional error than *F*_z_. Therefore, *F*_y_ was applied horizontally to only one point in the center of the FP direction to avoid applying *M_z_*. The force-applied position in the gravity direction is shown in Figure 8c. A photograph of the calibration setup is shown in Figure 8d. To apply only *F*_y_, a force gauge jig that enabled the force gauge to move only in the *y*-direction was fabricated by a 3D printer (Makerbot Replicator+, Stratasys, Eden Prairie, MN, USA). The FP and force gauge jig were fixed on the same stage to prevent skidding.

**Figure 9:**
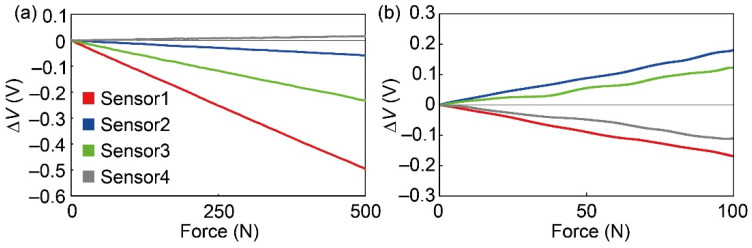
(**a**) Output voltage when a *z*-directional force is applied to A6. (**b**) Output voltage when a *y*-directional force is applied.

Figure 9a shows the relationship between *F*_z_ and the sensor output when *F*_z_ is applied to A6. We evaluated the gradient of each sensor output to *F*_z_, which corresponds to *S_ik_* in Equation (8). As in Equation (8), to obtain the calibration matrices *A_iM_*_x_ and *A_iM_*_y_ in Equation (2), we evaluated the gradient of each sensor output to *M*_x_ and *M*_y_, respectively. Figure 9b shows the relationship between *F*_y_ and the sensor output. *F*_y_ is expressed by the following equation using the gradient *s*_1_–*s*_4_ of the sensor element, *F*_y_ and pseudo-inverse matrix s*.

(9) $$\Delta V=\left(\begin{array}{cccc} s_{1} & s_{2} & s_{3} & s_{4} \end{array}\right)F_{y}=sF_{y}$$

(10) $$F_{y}=s^{*}\Delta V.$$

This s* is the calibration matrix of *F*_y_. From the above, calibration matrix ***A*** in Equation (2) is calculated as follows:

(11) $$A=\left[\begin{array}{cccc} -1899 & 2026 & 1391 & -1285\\ -633 & -750 & -605 & -643\\ -52.4 & 64.0 & -49.2 & 51.0\\ -127 & -151 & 126 & 130 \end{array}\right].$$

The units of calibration matrix ***A*** are N/V for the top two rows and N·m/V for the bottom two rows. The above equation confirms that the experimental calibration matrix matches Table 2 in the positive and negative directions. The cosine similarity of the calibration matrix is as follows:

(12) $$T=\left[\begin{array}{cccc} 0 & 94 & 75 & 94\\ & 0 & 97 & 84\\ & & 0 & 95\\ & & & 0 \end{array}\right].$$

The maximum separation became 97° between *F*_z_ and *M*_x_, and the minimum separation was 75° between *F*_y_ and *M*_x_. These values were sufficient to separate each axis. Based on the 80-Hz low-pass-filtered noise data, the resolution became *F*_y_: 1.9 N, *F*_z_: 1.4 N, *M*_x_: 0.054 N/m, and *M*_y_: 0.15 N/m.

**Figure 10:**
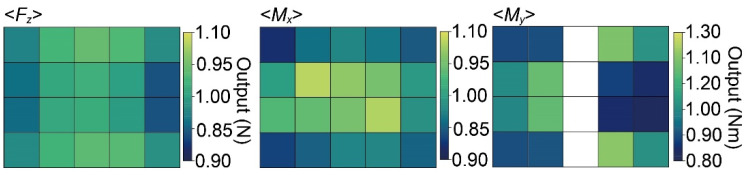
Positional errors in *F*_z_, *M*_x_, and *M*_y_, calculated by the measured data.

Figure 10 shows the output calculated by Equation (2) when 1 N or 1 Nm was applied to each point. The positional error for *F*_z_ was −6% to +4%; for *M*_x_, it was −8% to +8%; for *M*_y_, it was −17% to +21%. The output *F*_z_ and *M*_x_ were lower at a distance from the center than when the force was applied to the center. Additionally, the output *F*_z_ was lower for the distance from the center in the *x*-direction than in the *y*-direction. As shown in Figure 3, this tendency is identical to that in the simulation results. Likewise, the output *M*_x_ was lower for the distance in the *y*-direction than in the *x*-direction.

### 4.3. Walking Experiment

**Figure 11:**
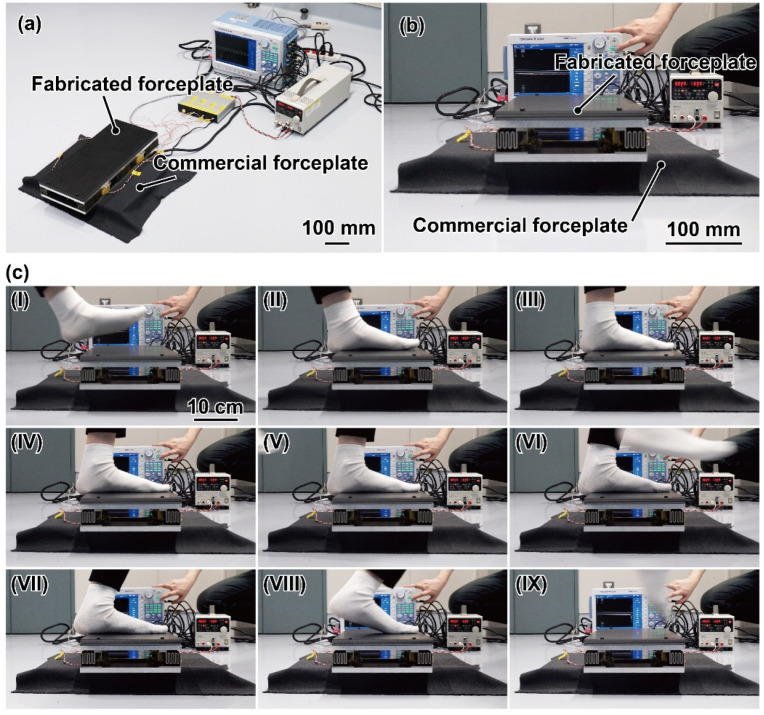
(**a**,**b**) Photographs of the experimental setup to demonstare the GRF measumement during walking. (**c**) Camera image during the experiment. The time interval is 0.1 ms. (**cI**) Camera image before the sole contacts the FP. (**cII**–**cVIII**) Camera image while FP is in contact with the sole. (**c-IX**) Camera image after the sole contacts the FP.

As in the demonstration, GRF during walking was measured by the fabricated FP. The subject was a healthy woman in her twenties with a body mass of approximately 50 kg. The experimental setup is shown in Figure 11a. For comparison, the fabricated FP was placed on a commercial FP (TF-3040; 300 mm × 400 mm, Tekkugihan, Kyoto, Japan), as shown in Figure 11b. Both FPs were connected to the same oscilloscope, which was used in the calibration. During the experiment, the sampling rate was 2000 Hz with a low-pass filter of 80 Hz. The FP was installed with the walking direction in the *y*-direction. Camera images with a time interval of 0.1 s while walking on the FP are shown in Figure 11c. As shown in the camera images, we measured the GRF of the left foot with a sock, which was a few steps after the start of walking. Based on the 80-Hz low-pass-filtered noise data, the resolution of the commercial FP became *F*_y_: 0.053 N, *F*_z_: 0.078 N, *M*_x_: 0.013 N/m, and *M*_y_: 0.0076 N/m.

**Figure 12:**
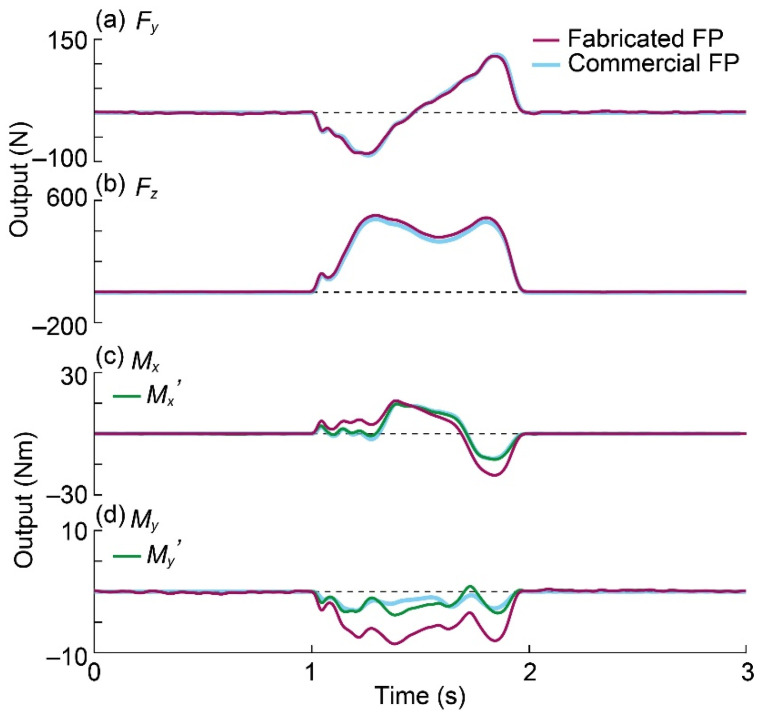
Measured GRF by the fabricated FP and commercial FP. (**a**) *F_y_*; (**b**) *F_z_*; (**c**) *M*_x_; (**d**) *M_y_*.

The GRF data obtained with the fabricated and commercial FPs are shown as purple and blue lines in Figure 12. The forces and moments obtained with the fabricated FP are defined as *F*_y*fab*_, *F*_z*fab*_, *M*_x*fab*_, and *M*_y*fab*_, and the forces and moments obtained with the commercial FP are defined as *F*_y*com*_, *F*_z*com*_, *M*_x*com*_, and *M*_y*com*_, respectively. Due to the force balance, *F*_y*fab*_, *F*_z*fab*_, and *F*_y*com*_, *F*_z*com*_ should have identical values. Meanwhile, *M*_x*fab*_, *M*_y*fab*_, *M*_x*com*_, and *M*_y*com*_ are not equal because the center points of both FPs are in different positions. Therefore, to compare the fabricated FP and commercial FP, the moments *M*_x*fab*_ and *M*_y*fab*_ must be transformed into the moments around the central point of the commercial FP. The transformed *M*_x*fab*_ and *M*_y*fab*_ are denoted by *M*_x_*’* and *M*_y_*’* and are shown as green lines in Figure 12c,d.

The *x*-axis moment around the center point of the commercial FP from the output of the fabricated FP is expressed in the following equation using fabricated height *H* of the FP. The moment in the *y*-axis of the fabricated FP can also be converted to a moment around the center point of the commercial FP using *F*_x*fab*_; however, this is omitted because *F*_x_ in normal walking is small.

(13) $$M_{x}^{\prime }=M_{x_{fab}}+\left(H\times F_{y_{fab}}\right).$$

In this experiment, the center points of the FPs were displaced by approximately 1 cm in the *x*-direction. Therefore, the moment in the *y*-axis around the commercial FP center point was calculated from the output obtained with the fabricated FP and is expressed by the following equation:

(14) $$M_{y}^{\prime }=M_{y_{fab}}+\left(0.01\times F_{z_{fab}}\right).$$

In the case of *F*_y_, the difference between *F*_y*fab*_ and *F*_y*com*_ became 2.4% for the first negative peak and 4.4% for the second positive peak. In the case of *F*_z_, the difference was 5.1% and 5.9% in the first and second positive peaks, respectively. Additionally, the cosine similarities between *F*_y*fab*_ and *F*_y*com*_, *F*_z*fab*_ and *F*_z*com*_, *M*_x_*′* and *M*_x*com*_, *M*_y_*′* and *M*_y*com*_ became 3.3°, 1.0°, 4.8°, and 21°, respectively. Thus, *F*_z_, *F*_y_, and *M*_x_ were considered sufficiently consistent with the fabricated FP and commercial FP. Meanwhile, *M*_y_ was not sufficiently consistent. This disagreement is thought to occur because of the misalignment of the FP installation and the small value of *M*_y_.

During normal walking, GRF in the y-direction is applied in the opposite direction when heeling contacts and the forward direction when the toe kicks. The GRF in the *z*-direction shows a bimodal waveform with two peaks and a valley [33]. According to Figure 12a,b, *F*_y*fab*_ and *F*_z*fab*_ have identical characteristics to the time variation in the *y*-direction and *z*-direction of normal walking. During normal walking on a plane, the GRF is approximately ±20% and +120% of the body weight in the front–back and vertical directions, respectively [33,34]. In the current study, the minimum *F*_y*fab*_ of the measured GRF was −83 N (−16.6%), the maximum *F*_y*fab*_ was 116 N (23.2%), and the maximum *F*_z*fab*_ was 503 N (100.6%). Thus, we consider the GRF value to be reasonable, and we can measure the GRF of a normal walking with the fabricated FP.

Our fabricated FP can theoretically be used by being implanted into a staircase; even the vertical GRF increases by approximately 150% of the body weight for stair descent on large inclination stairs of 42.0° [26]. The fabricated FP was designed to fit the major run length of Japanese residential stairs, which is 200–240 mm. This design lies within the regulations in Japan but not those of other countries [25]. The GRF during stair walking is generally larger on stairs with a larger inclination. Since Japanese regulations are the laxest and permit stairs with a larger inclination, the maximum GRF value can be tested based on Japanese regulations. However, additional experiments on stairs with several sizes of FP will be necessary in the future since the FP performance will change when the size of the FP changes. At present, the developed FP has a lower resolution; however, the resolution will be enhanced by improving the measurement circuit. The proposed FP also measured two-directional forces and two-directional moments. In principle, we can design an FP, which detects three-dimensional forces and three-directional moments, by changing the spring property and adding other sensor elements for the x-axis force detection.

## 5. Conclusions

In this research, we proposed an FP that was composed of mechanical springs and noncontact displacement sensors. Since the sensor elements do not affect the mechanical properties of the FP, the FP performance was determined by the spring design. The FP was designed to be 200 mm × 450 mm × 67 mm, to measure the GRF during walking on indoor corridors or stairs. The resonant frequency of the fabricated FP was over 100 Hz, which was sufficiently high to allow accurate measurement of the GRF during human walking. The force applied to the plate surface was calculated via a linear sum of the sensor outputs with the calibration matrix. It was confirmed that the FP could measure forces and moments in two directions with good independence. Finally, we demonstrated that the fabricated FP measured the GRF of humans walking similarly to the commercial FP. Therefore, the proposed FP method, which enables easy modification of the shape and performance, is useful for GRF evaluation.

## Figures and Tables

**Table 1:** Parameters of the position and size dimension of the coil, magnet, spring, and plate.

	*h*	*w*	*l*_1_	*l*_2_	*s*	*t*	*φ*	*g*	*l*_m_
Length (mm)	200	450	43	80	24	120	10	3	20

**Table 2:** Positive and negative directions of the sensor outputs when each directional force and moment are applied.

	*F*_x_	*F*_y_	*F*_z_	*M*_x_	*M*_y_
Sensor 1	NA	−	−	−	−
Sensor 2	NA	+	−	+	−
Sensor 3	NA	+	−	−	+
Sensor 4	NA	−	−	+	+

## Data Availability

The data presented in this study are available on request from the corresponding author.

## References

[1] Akashi P.M.H., Sacco I.C.N., Watari R., Hennig E. (2008). The effect of diabetic neuropathy and previous foot ulceration in EMG and ground reaction forces during gait. Clin. Biomech..

[2] Stacoff A., de Quervain I.A.K., Luder G., List R., Stüssi E. (2007). Ground reaction forces on stairs. Part II: Knee implant patients versus normals. Gait Posture.

[3] Begg R.K., Sparrow W.A., Lythgo N.D. (1998). Time-domain analysis of foot-ground reaction forces in negotiating obstacles. Gait Posture.

[4] Castro M., Abreu S., Sousa H., Machado L., Santos R., Vilas-Boas J.P. (2013). Ground reaction forces and plantar pressure distribution during occasional loaded gait. Appl. Ergon..

[5] Cavanagh P.R., Lafortune M.A. (1980). GRF in distance running. J. Biomech..

[6] Hong H., Kim S., Kim C., Lee S., Park S. (2013). Spring-like gait mechanics observed during walking in both young and older adults. J. Biomech..

[7] Mei Q., Fernandez J., Fu W., Feng N., Gu Y. (2015). A comparative biomechanical analysis of habitually unshod and shod runners based on a foot morphological difference. Hum. Mov. Sci..

[8] Kram R., Powell A.J. (2012). A treadmill-mounted force platform A treadmill-mounted force platform. J. Appl. Physiol..

[9] Masani K., Kouzaki M., Fukunaga T. (2002). Variability of ground reaction forces during treadmill walking. J. Appl. Physiol..

[10] Jansen E.C., Vittas D., Hellberg S., Hansen J. (1982). Normal gait of Young and old men and women: Ground reaction force measurement on a treadmill. Acta Orthop..

[11] Park J., Na Y., Gu G., Kim J. (2016). Flexible insole ground reaction force measurement shoes for jumping and running. Proceedings of the 6th IEEE/RAS-EMBS International Conference on Biomedical Robotics and Biomechatronics.

[12] Liedtke C., Fokkenrood S.A.W., Menger J.T., van der Kooij H., Veltink P.H. (2007). Evaluation of instrumented shoes for ambulatory assessment of ground reaction forces. Gait Posture.

[13] Heglund B.Y.N.C. (1981). Short Communication Force-Plate to Measure Ground Reaction Forces. J. Exp. Biol..

[14] Ferryanto F., Akbar M.M., Wicaksono S., Mahyuddin A.I. (2021). Design, Manufacture, and Testing of a Low-Cost Force Platform with 3-Axis Load Cell. IOP Conference Series: Materials Science and Engineering.

[15] Silva M.G., Moreira P.V.S., Rocha H.M. (2017). Development of a low cost force platform for biomechanical parameters analysis. Res. Biomed. Eng..

[16] Ogawa A., Mita A., Yorozu A., Takahashi M. (2017). Markerless knee joint position measurement using depth data during stair walking. Sensors.

[17] Liikavainio T., Isolehto J., Helminen H.J., Perttunen J., Lepola V., Kiviranta I., Arokoski J.P.A., Komi P.V. (2007). Loading and gait symmetry during level and stair walking in asymptomatic subjects with knee osteoarthritis: Importance of quadriceps femoris in reducing impact force during heel strike?. Knee.

[18] Ackermans T.M.A., Francksen N.C., Casana-Eslava R.V., Lees C., Baltzopoulos V., Lisboa P.J.G., Hollands M.A., O’Brien T.D., Maganaris C.N. (2019). A novel multivariate approach for biomechanical profiling of stair negotiation. Exp. Gerontol..

[19] Ackermans T.M.A., Francksen N.C., Casana-Eslava R.V., Lees C., Baltzopoulos V., Lisboa P.J.G., Hollands M.A., O’Brien T.D., Maganaris C.N. (2020). Stair negotiation behaviour of older individuals: Do step dimensions matter?. J. Biomech..

[20] Ackermans T., Francksen N., Lees C., Papatzika F., Arampatzis A., Baltzopoulos V., Lisboa P., Hollands M., O’Brien T., Maganaris C. (2021). Prediction of Balance Perturbations and Falls on Stairs in Older People Using a Biomechanical Profiling Approach: A 12-Month Longitudinal Study. J. Gerontol.-Ser. A Biol. Sci. Med. Sci..

[21] Wang K., Delbaere K., Brodie M.A.D., Lovell N.H., Kark L., Lord S.R., Redmond S.J. (2017). Differences between Gait on Stairs and Flat Surfaces in Relation to Fall Risk and Future Falls. IEEE J. Biomed. Health Inform..

[22] Foster R.J., Maganaris C.N., Reeves N.D., Buckley J.G. (2019). Centre of mass control is reduced in older people when descending stairs at an increased riser height. Gait Posture.

[23] Roys M.S. (2001). Serious stair injuries can be prevented by improved stair design. Appl. Ergon..

[24] Novak A.C., Komisar V., Maki B.E., Fernie G.R. (2016). Age-related differences in dynamic balance control during stair descent and effect of varying step geometry. Appl. Ergon..

[25] Ogawa A., Iijima H., Takahashi M. (2021). Staircase design for health monitoring in elderly people. J. Build. Eng..

[26] Riener R., Rabuffetti M., Frigo C. (2002). Stair Ascent and Descent at Different Inclinations. Gait Posture.

[27] Zumwalt A.C., Hamrick M., Schmitt D. (2006). Force plate for measuring the ground reaction forces in small animal locomotion. J. Biomech..

[28] Wilson A.M., Seelig T.J., Shield R.A., Silverman B.W. (1998). The effect of foot imbalance on point of force application in the horse. Equine Vet. J..

[29] Stacoff A., Diezi C., Luder G., Stüssi E., Kramers-De Quervain I.A. (2005). Ground reaction forces on stairs: Effects of stair inclination and age. Gait Posture.

[30] Christina K.A., Cavanagh P.R. (2002). Ground reaction forces and frictional demands during stair descent: Effects of age and illumination. Gait Posture.

[31] Ruben R.J. (2000). Special communication. Int. J. Pediatr. Otorhinolaryngol..

[32] Villeger D., Costes A., Watier B., Moretto P. (2014). An algorithm to decompose ground reaction forces and moments from a single force platform in walking gait. Med. Eng. Phys..

[33] Marasovič T., Cecič M., Zanchi V. (2009). Analysis and interpretation of ground reaction forces in normal gait. WSEAS Trans. Syst..

[34] Keller T.S., Weisberger A.M., Ray J.L., Hasan S.S., Shiavi R.G., Spengler D.M. (1996). Relationship between vertical ground reaction force and speed during walking, slow jogging, and running. Clin. Biomech..

